# Herpetic endotheliitis and stromal keratitis following inactivated COVID‐19 vaccination

**DOI:** 10.1002/ccr3.6397

**Published:** 2022-10-06

**Authors:** Mehrdad Mohammadpour, Hossein Farrokhpour, Reza Sadeghi

**Affiliations:** ^1^ Translational Ophthalmology Research Center Farabi Eye Hospital, Tehran University of Medical Sciences Tehran Iran

**Keywords:** COVID‐19, endotheliitis, herpetic keratitis, HSV, vaccination

## Abstract

Given the scarcity of data regarding ocular complications following COVID‐19 vaccination, the approach toward patients with suggestive symptoms and established clinical practice is lacking. Herein, we report the first case of herpetic endotheliithis and secondary stromal keratitis following inactivated COVID‐19 vaccination who experienced a relapse due to poor adherence.

## Graphical abstract

Although the causality between COVID‐19 vaccination and herpetic keratitis cannot be established, this case report highlights the need for practitioners to be mindful of the possibility of herpetic keratitis reactivation in patients with suggestive symptoms and previous COVID‐19 vaccination and urges the need for proper follow‐up and treatment.

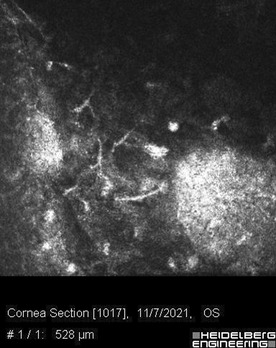

## 1 INTRODUCTION

The wide use of vaccines with various mechanisms against Corona Virus 2019 (COVID‐19) has given us relatively high control over the ongoing pandemic.^1^ However, due to the unprecedented rate of vaccine utilization, the adverse events associated with them remain yet to be fully elucidated.^2^ The ocular complications following COVID‐19 vaccine administration are among the manifestations reported mainly through case reports and series.^3^ In the most recent study examining adverse events in patients receiving Food and Drug Administration‐approved vaccines, it was demonstrated that patients in the age groups of 40–59, 30–39, and 70–79 had the highest rates of ocular side effects reported. Additionally, it was shown that “Eye swelling, ocular hyperemia, conjunctivitis (33.33%), blurred vision (26.69%), and visual impairment (19.77%) had the highest frequency among reported symptoms.^4^ Wang et al., using Reported In Population‐Based Pharmacovigilance Surveillance Systems, revealed that ocular inflammatory diseases, including uveitis, followed by optic neuropathy, anterior segment, and posterior segment yielded the highest prevalence, respectively.^5^ In another study, involvement of the anterior segment was reported among the second most prevalent complications among all vaccine types.^6^ Although the reactivation of herpes simplex virus 1 (HSV‐1) and herpes zoster virus (HZV) resulting in herpetic keratitis and herpes zoster ophthalmicus following COVID‐19 vaccine administration has been reported in some sporadic case reports,^3^, ^7^, ^8^, ^9^ occurrence of herpetic keratitis endotheliitis preceded by the Sinopharm/BBIBP vaccine (inactivated virus platform) has not been described elsewhere. Herein, we set to report a case of HSV‐1 reactivation following the Sinopharm vaccine and provide the available data and our approach toward the case.

## 2 CASE HISTORY/EXAMINATION

**FIGURE 1:**
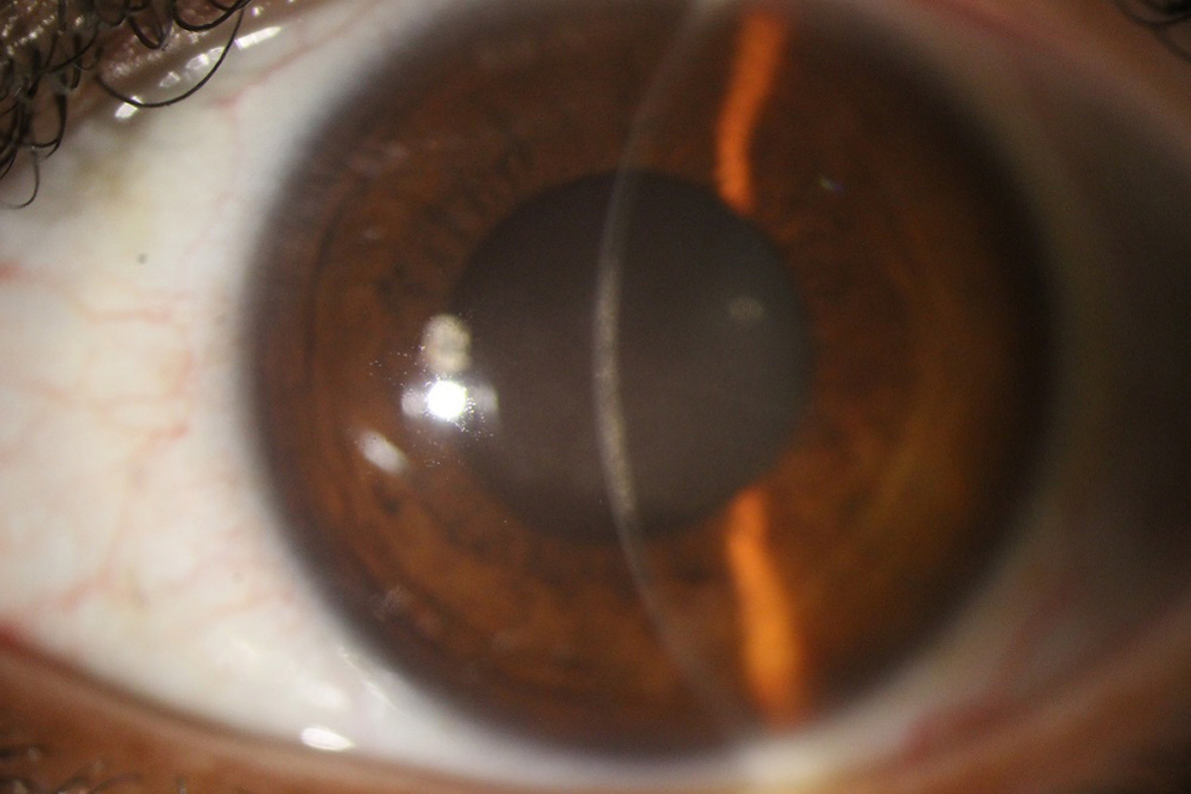
Slit lamp anterior segment image indicating central corneal opacity

**FIGURE 2:**
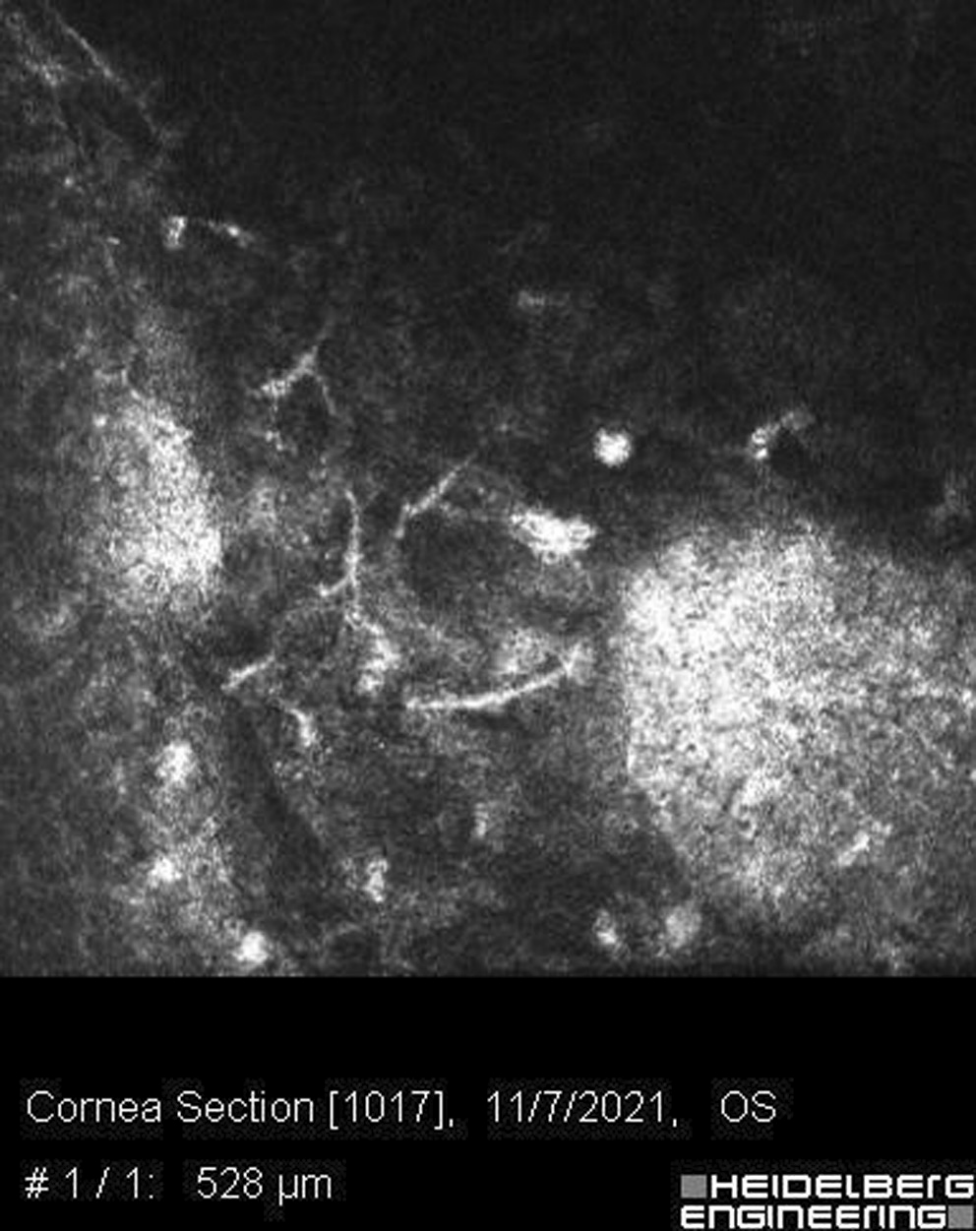
Confocal microscopy exam of the left eye, dentate infiltration in the endothelial layer of cornea

**FIGURE 3:**
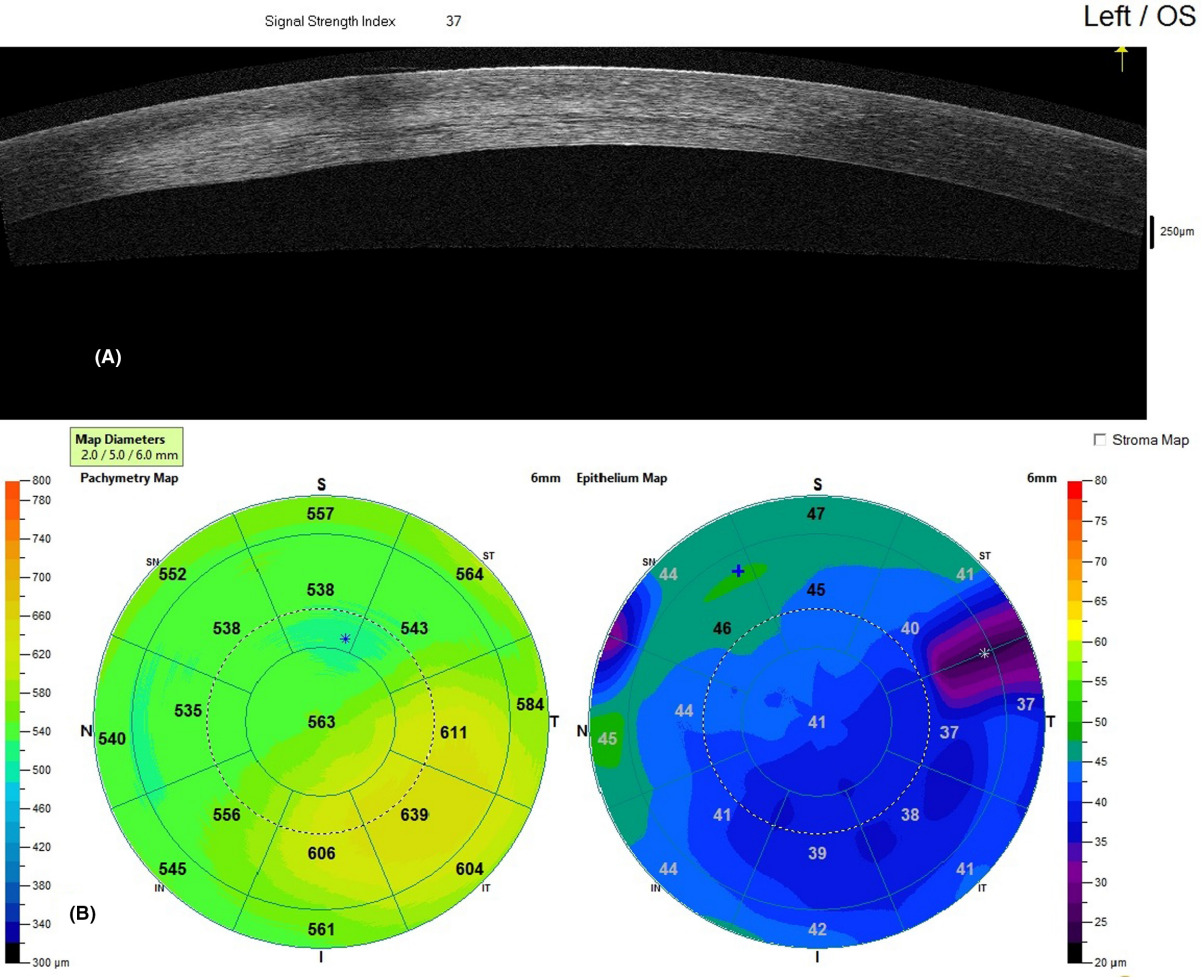
Left eye anterior segment optical coherence tomography, (A) strength signal index in the anterior segment indicating pathological changes across endothelial and stromal layers. (B) Stromal thickening accompanied by epithelial thinning on the stromal map

A 30‐year‐old woman, a known case of hypothyroidism with regular levothyroxine use, presented to the Farabi Eye Hospital, a tertiary eye center in Iran, with reduced visual acuity in her left eye for 3 weeks prior to her referral. She reported receiving the Sinopharm vaccine 2 weeks before her symptoms, which were not accompanied by systemic manifestations except soreness at the injection site for 1 day. She sought medical help when the symptoms started. She was prescribed acyclovir 400 mg, three times daily, and Fluorometholone eye drop, one drop three times daily by another ophthalmologist in the private section. However, therapy was discontinued after 2 weeks at the patient's discretion after subsiding initial symptoms. Other than the worsened vision, there was no report of other ocular symptoms, including pain, redness, and photophobia, at the time of presentation to the Farabi eye hospital. Her past ocular history before vaccination was unremarkable, and there was no report of previous episodes of herpetic keratitis previous to vaccination. On the examination, her extra‐ocular examination was normal. Motility was intact. Pupils' examination revealed that they were equally round and reactive to light and accommodation. Visual acuity values were 10/10 OD and 9/10 OS using a Snellen chart. Intraocular pressure on digital tonometry was 14 and 15 mmHg in OD and OS, respectively. Conjunctiva and sclera had no significant pathological findings, and no injection was observed. On the anterior segment examination, pupils were round, regular, and reactive. The swinging light test was negative for relative afferent pupil defect. The cornea thickness was normal. The cornea in the left eye showed a central opacity measuring3 × 3 mm and stromal infiltration under slit‐lamp microscopy (Figure 1). Iiris's examination showed no sign of defection. In the posterior segment examination, no sign of inflammation was observed. Normal foveal reflex was detected. The optic nerve head was round, sharp, and pink with a cup to disc ratio of 3/10, and the peripheral retina was attached. Dilated fundus examination was normal. No hemorrhagic or other abnormalities were notable. Basic fluorescein staining was normal, and no dendritic ulcer or other characteristics of HSV epithelial keratitis was observed. The confocal scan revealed disciform endothelial infiltrates on endothelial and stromal surface indicative of herpetic endotheliitis and secondary stromal keratitis. (Figure 2) Furthermore, anterior segment optical coherence tomography showed epithelial thinning and stromal thickening. (Figure 3).

## 3 DIFFERENTIAL DIAGNOSIS, INVESTIGATIONS, AND TREATMENT

The patient had a history of prescription of acyclovir 400 mg three times daily and Fluorometholone eye drop one drop three times daily. However, it was discontinued after 2 weeks at the patient's discretion after subsiding initial symptoms, resulting in a relapse in the keratitis and endotheliitis with blurred vision. Consequently, the medication was switched to oral valacyclovir 1 g, twice daily for 14 days, followed by a maintenance dose of 1 g daily for the rest of the follow‐up period and a corticosteroid (betamethasone) eye drop every 3 h. The patient was then reevaluated daily.

## 4 OUTCOME AND FOLLOW‐UP

At the 10 days mark from the beginning of the new treatment, the visual acuity in the left eye improved to 10/10. Our evaluation after 3 months showed marked treatment of corneal opacity, and the patient's symptoms were gone, although the patient continued to receive the treatment at a lower dose.

## 5 DISCUSSION

Several potential ocular adverse events following various vaccines have been reported in the literature, with the eyelids and conjunctiva being the most involved area. Accordingly, more limited incidents following COVID‐19 vaccines have been observed. In a case series by Pichi et al., 9 patients with ocular complications, including 2 patients with anterior scleritis and 2 patients with acute macular retinopathy, were reported.^10^ In other reports, including 2 systematic reviews, various ocular manifestations involving different parts of the eye, including cornea, eyelids, conjunctiva, and retina, following COVID‐19 vaccination were described.^3^, ^11^

The reactivation of HSV‐1 and VZV has also been reported. Richardson‐May et al. described an 82‐year‐old man with a previous history of HSV‐1 who experienced recurrence 1‐day post‐vaccination with AstraZeneca and was diagnosed with herpetic stromal keratitis.^9^ Alkhalifah et al. reported 2 cases with a previous history of herpes, vaccinated with Pfizer‐BioNTech mRNA vaccine who developed necrotizing stromal keratitis and endotheliitis and epithelial keratitis, respectively.^7^ Four cases vaccinated with either Oxford‐AstraZeneca or Pfizer‐BioNTech who developed herpetic keratitis and corneal endotheliitis 1 week after the second dose were remarked by Alkwikbi et al.^8^ The time frame from the vaccination to the presentation to the emergency room across the respected reports varied from 4 days to 4 weeks. Although almost all of the mentioned patients experienced recovery from the keratitis, residual scarring and persistent epithelial inflammation remained in some cases. Noticeably, the history of prior HSV‐1 presentation was absent in some cases. While the Sinopharm vaccine utilizes attenuated COVID‐19 virus to induce immunogenicity, the mRNA vaccines achieve immunogenicity through the introduction of the Spike protein gene into the host cells, leading to production of spike proteins and eventually their presentation to the immune system.^12^ In a recent study, it was revealed that that Sinopharm vaccine has relatively lower performance in terms of producing IgG antibodies and other parameters as well.^13^ In return, the higher immune response could trigger unintended adverse reactions through immune enhancement in various parts of body. This explains the relatively lower inflammatory adverse events including ocular ones, reported after inactivated vaccines.^14^

Although several mechanisms that could potentially contribute to the reactivation of HSV‐1 after vaccination have been postulated, the definitive insight into the responsible mechanism is still needed to be elaborated. Auto inflammation as a result of attenuation of the immune regulatory responses and cytokine production, molecular mimicry of pathogen particles, shifting the focus of immunologic surveillance on the vaccination site, and the ensuing lowered immunologic potency are among these theories which have the potential to cause the recurrence. It has been shown that the toll‐like receptors (TLRs) play an imperative role in starting the innate immune response by HSV recognition and in the virus maintenance in the body. Furthermore, T helper (Th) 1 cells have been recognized as one of the main contributors to the initiation and progression of HSV keratitis. In contrast, Th2 cells have been primarily associated with alleviating recurrence^15^.^16^ Although all these mechanisms are affected following vaccination,^17^ there is a high debate regarding the mixed effect of Th1 and Th2 cells in the promotion of or against the inflammatory cascade after COVID‐19 vaccination, given the inflicting results.^18^ On the contrary, the evidence concerning the role of T helper 17 cells in deriving the immune system toward an inflammatory state following both infections with COVID‐19 and vaccination, possibly through immune enhancement by interleukin 6 induction, has been mostly uniform.^18^ Interestingly, the essential effect of Th‐17 cells in deriving an immune state into a hyperinflammatory response during HSV stromal keratitis and herpes zoster reactivation has been established.^19^, ^20^ This association could, in theory, have a possible contribution to the reactivation of HSV after vaccination.

## 6 CONCLUSION

To the best of our knowledge, this is the first report of herpetic endotheliitis and stromal keratitis following the Sinopharm/BBIBP vaccine. Although the temporal link between vaccination and adverse ocular events, including the HSV‐1 reactivation, cannot be established through these case reports, these reports add to the sparse body of evidence supporting this notion. Additionally, it necessitates bearing in mind the possibility of herpetic keratitis when facing patients with suggested symptoms and previous vaccination. Finally, the decision regarding the preventive therapy for HSV‐1 and VZV recurrence needs further evidence to be addressed in detail.

## AUTHOR CONTRIBUTIONS

Mehrdad Mohammadpour: conception and design, writing—original draft, critically revising the manuscript. Hossein Farrokhpour: conception and design, writing—original draft, critically revising the manuscript. Reza Sadeghi: analysis and interpretation of data, writing—revising the original draft critically, review the revised version. All authors who contributed to this study read and approved the final version of the revised manuscript to be published and agreed to be responsible for all aspects of the work.

## FUNDING INFORMATION

No funds from the private or public sector were received for this study.

## CONFLICT OF INTEREST

The authors of this study declare that they have no conflict of interest.

## ETHICAL APPROVAL

This study's protocol is in line with the 2013 Helsinki declaration and approved by the Ethics Committee of Tehran University of Medical Sciences.

## CONSENT

Written informed consent was obtained from the patient to publish this report in accordance with the journal's patient consent policy.

## Data Availability

The data used in the current study is available upon reasonable request
